# Alcohol-Related Context Modulates Performance of Social Drinkers in a Visual Go/No-Go Task: A Preliminary Assessment of Event-Related Potentials

**DOI:** 10.1371/journal.pone.0037466

**Published:** 2012-05-17

**Authors:** Géraldine Petit, Charles Kornreich, Xavier Noël, Paul Verbanck, Salvatore Campanella

**Affiliations:** Laboratory of Psychological Medicine, Free University of Brussels, Brussels, Belgium; University of Granada, Spain

## Abstract

**Background:**

Increased alcohol cue-reactivity and altered inhibitory processing have been reported in heavy social drinkers and alcohol-dependent patients, and are associated with relapse. In social drinkers, these two processes have been usually studied separately by recording event-related potentials (ERPs) during rapid picture presentation. The aim of our study was to confront social drinkers to a task triggering high alcohol cue-reactivity, to verify whether it specifically altered inhibitory performance, by using long-lasting background picture presentation.

**Methods:**

ERP were recorded during visual Go/No-Go tasks performed by social drinkers, in which a frequent Go signal (letter “M”), and a rare No-Go signal (letter “W”) were superimposed on three different types of background pictures: neutral (black background), alcohol-related and non alcohol-related.

**Results:**

Our data suggested that heavy social drinkers made more commission errors than light drinkers, but only in the alcohol-related context. Neurophysiologically, this was reflected by a delayed No-Go P3 component.

**Conclusions:**

Elevated alcohol cue-reactivity may lead to poorer inhibitory performance in heavy social drinkers, and may be considered as an important vulnerability factor in developing alcohol misuse. Prevention programs should be designed to decrease the high arousal of alcohol stimuli and strengthen cognitive control in young, at-risk individuals.

## Introduction

Response inhibition, defined as the ability to suppress inappropriate thoughts and actions, is an important component of human behavior. Indeed, deficits in inhibitory control have been reported in several pathological states, such as euthymic bipolar disorder and Huntington's disease [1]–[2]. This lack of inhibition has been widely demonstrated using “Go/No-Go” tasks, based on the suppression of a prepotent “Go” response. Two main event-related potential (ERP) components have been investigated, i.e., N2 and P3, as it is common to observe a larger frontal N2 and frontocentral P3 on inhibition trials [3]–[5]. The functional significance of these components remains a matter of debate. However, the N2 is thought to reflect conflict monitoring and effortful processing, involving mainly the rostral anterior cingulate cortex [6]–[8], while the P3 is thought to reflect the inhibition function per se, involving mainly the ventrolateral prefrontal cortex [6], [8]–[9].

Interestingly, altered inhibitory processes have been suggested in patients with alcohol dependence. Indeed, Kamarajan *et al.* revealed a lower P3 amplitude in the No-Go conditions (but normal N2 amplitude) in alcoholics and their offspring, indicating that lower No-Go P3 amplitudes were associated with inhibitory alterations and thus vulnerability to alcohol [10]–[11]. These and other studies led scientists to consider alcoholism as a “disinhibitory disorder,” associated with impaired control over automatic impulses to drink [12]. Moreover, alcoholics exhibit a basic prepotent response inhibition deficit, which is enhanced when the response to be suppressed is related to alcohol [13]. Indeed, conditioned appetitive responses to alcohol cues may reinstate alcohol-seeking behavior, and may induce relapse even after long periods of abstinence [14]–[16]. The visual presentation of alcohol (*vs.* abstract cues) induces the activation of the brain reward system in regions such as the ventral striatum, the orbitofrontal cortex and the dorsal anterior cingulate cortex, which play a role in reward-based decision-making [17]. Interestingly, converging evidence also came from non-brain imaging (ERP, fMRI) studies, such as the study by Miller and Fillmore [18], which showed that social drinkers display greater eye fixation times towards alcohol-related stimuli than neutral images, even under high doses of alcohol.

These data constituted the base for dual-process theories, which suggested that there are two processes associated with drinking behavior: (1) an automatic process characterized by an increase in the salience of alcohol-related cues, which tend to “grab the attention” of experienced drinkers; and (2) a lack of executive resources needed to inhibit the salient and dominant response, i.e., to drink due to the neurotoxic effects of repeated alcohol consumption and/or a state of vulnerability [19]–[20]. From a clinical standpoint, it is critical to identify how the imbalance of these two systems can predict relapse, to optimize alcohol intervention by the healthcare system [16].

Similar to alcoholics, heavy social drinkers display decreased performance, increased urge to drink and elevated P3 while exposed to alcohol cues than light drinkers [21]–[26]. They also present a decreased No-Go P3 (but normal No-Go N2) in a Go/No-Go task with letters [9]. In this regard, the effect of exposure to alcohol-related cues on inhibitory processes in social drinkers should be further investigated. Indeed, alcohol advertising is associated with subsequent alcohol consumption in young people, and young drinkers are especially at risk of developing alcohol dependence and loss of control over drinking, probably because of the attractive valence of alcoholic drinks [27]–[29]. The fact that social drinkers rarely reach the upper range of dependent criteria, because physical symptoms of withdrawal are unusual in this population [30], questions the relative importance of dependence severity and drinking patterns in alcohol cue reactivity. Thus, the identification of markers of vulnerability in young social drinkers at risk of alcohol misuse or alcoholism is important in developing adapted prevention programs.

All studies mentioned above compared light social drinkers and heavy drinkers, and explored alcohol cue-reactivity or inhibitory capacity, mainly reporting a significant modulation of the P3 amplitude. However, none directly investigated the interaction between these two processes. Moreover, these studies used brief visual presentations, such as letters and alcohol-related, neutral or arousing (erotic, adventure-related) slides [9], [24]–[26], although under natural conditions, controlling drinking behavior within long-term affective situations is often required [31]. Behavioral and neural reactions provoked by short-duration stimuli are clearly not as intense or complex as those generated by longer emotional contexts [32].

Therefore, in the present study, we aimed to confirm the hypothesis that long-term exposure to alcohol-related cues would reveal higher cue-reactivity and lower inhibitory capacity in heavy drinkers than in light drinkers. For this purpose, we examined the No-Go P3 and N2 components of heavy and light social drinkers performing a Go/No-Go task, in which a frequent Go signal (letter “M”), and a rare No-Go signal (letter “W”) were superimposed on three different types of context: neutral (black background picture), alcohol-related and non-alcohol-related. Moreover, recent findings suggested a strong association between cue-reactivity and relapse, subjective craving [33] and alteration of impulsive personality [34]. Therefore, subjective craving and impulsivity were also assessed, and the relation between these factors and errors of commission, as well as their associated ERP parameters, was also studied.

## Materials and Methods

### Study participants

An online prescreening survey was conducted among students of the Faculty of Psychology of the Free University of Brussels (Belgium) about their alcohol and drug consumption.

Alcohol abstainers were excluded from the study, as well as students with major medical problems, history of central nervous system disorders (including epilepsy and brain trauma), visual impairment, past or current drug consumption (other than alcohol) and family history of alcoholism. Subjects currently consuming cannabis (at least once in the month before the study) were not selected. There were nicotine users in both groups (low drinkers: n = 4/18; high drinkers: n = 6/17). The inclusion criteria were alcohol consumption since before starting university until inclusion in the study (mean years of regular alcohol consumption: low: 4.38 (1.68); high: 4.47 (1.87); p = 0.893; mean number of drinks per week consumed in the year before the experiment: low: 4.46 (3.66); high: 20.35 (9.74); p<0.001). Finally, 36 students between the ages of 18 and 25 were included, but one participant was later excluded because of too many probes with alpha-rhythm contamination.

The local ethics committee of the Brugmann Hospital (“Comité d'Ethique Hospitalier OM 026”) approved the study. Informed written consent to participate in the study after receiving full details regarding the aims and tests to be performed was obtained for all participants.

### Go/No-Go task

During the Go/No-Go tasks, the participants sat in a dark room on a chair placed one meter from a screen, and were instructed to press a button with the thumb of their right hand, as fast and accurately as possible, whenever the letter M (Go) was displayed, and to withhold pressing the button when the letter W (No-Go) was displayed [31].

**Figure 1:**
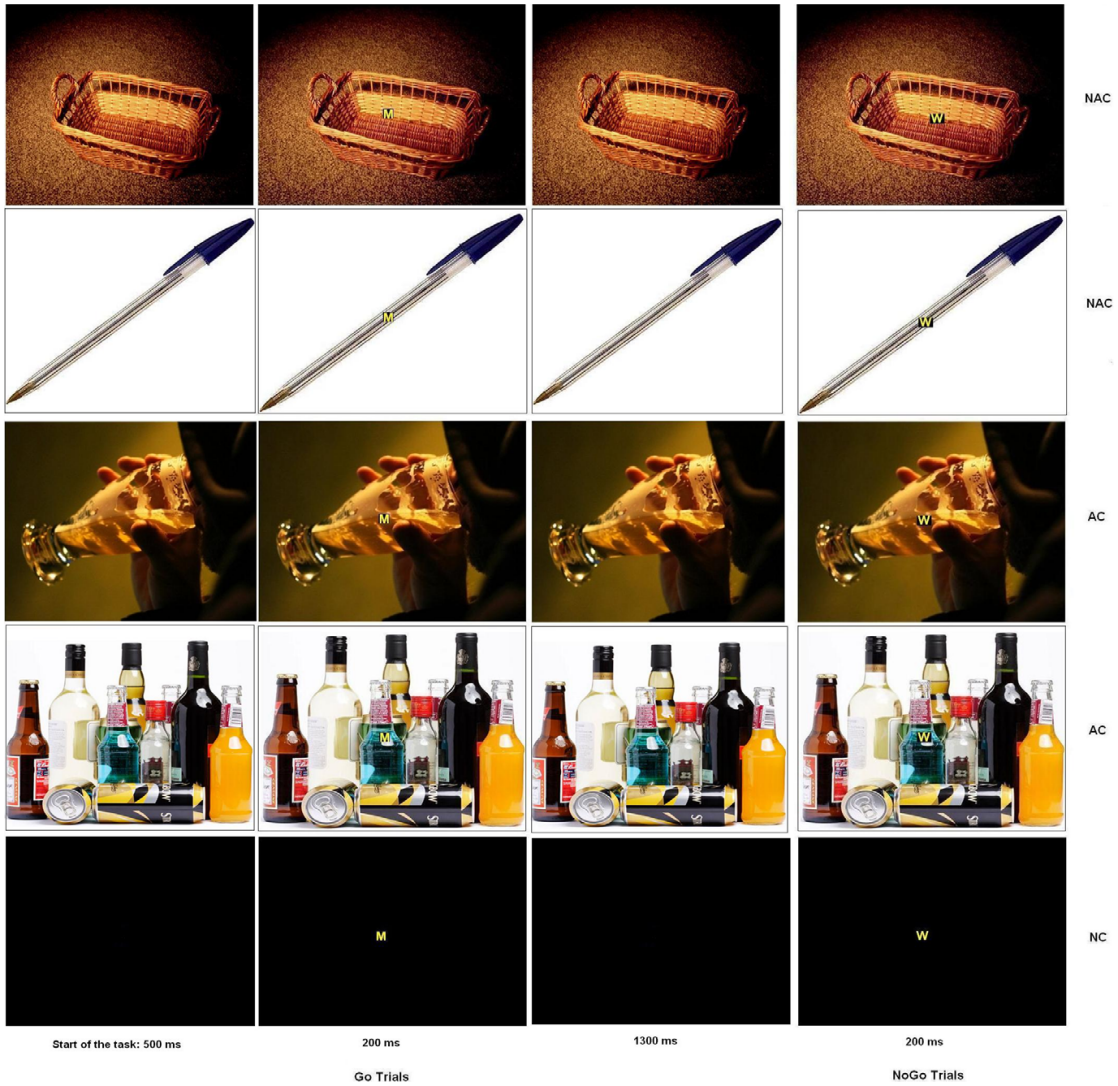
Go/No-Go task. Participants were confronted with six blocks of 133 stimuli, divided in 93 Go trials (letter M), and 40 No-Go trials (letter W). The letters were superimposed on two non-alcohol-related background pictures (NAC), two alcohol-related background pictures (AC) or a neutral black background (NC).

Both letters were superimposed on a background picture, conveying three different emotional contexts: neutral (black background; NC), alcohol (AC) and non-alcohol-related (NAC) (Fig. 1). Two different pictures were used for each of the alcohol and non-alcohol-related contexts. The black background was displayed twice, and the order in which the contexts were displayed was counterbalanced across participants.

Overall, the task comprised six separate stimulation blocks. Each block contained 133 letters, divided into 93 Go (70%) and 40 No-Go (30%) letters. Go and No-Go letters were displayed in a semi-random order to avoid the consecutive presentation of two No-Go letters within each block. One to four Go letters could precede No-Go letters. Each task consisted of the presentation of a background screen (black for NC, or AC or NAC; 500 ms), then the letter M or W appeared on this background screen for 200 ms, followed by a return to the initial background screen (1300 ms). Thus, subjects had a maximum of 1500 ms to press the button before the next letter appeared. Participants were asked to look at the center of the screen continuously and to refrain from moving and blinking during blocks to reduce interference caused by movements.

### Stimuli

The stimuli consisted of two capital letters (M and W; size of 500×400 mm) in Arial font and four background pictures (two alcohol-related and two non-alcohol-related) displayed on a 17-inch monitor. Participants were placed one meter from the screen. The letters were yellow with a black outline, to be clearly visible against the background.

First, 44 pictures were selected from the Internet and the International Affective Picture System, and equalized for brightness and net color with Photoshop 6.0 [35]. Then, 40 students who did not take part in the ERP study rated these pictures for alcohol-relatedness and emotional level: (1) for alcohol-relatedness, students were asked to rate whether the picture was strongly related to alcohol on a scale from zero (not at all) to five (extremely); (2) for emotional level, students were asked to rate how pleasant the picture was on a scale from zero (very unpleasant) to nine (very pleasant).

Finally, the two pictures with the lowest ratings on alcohol-relatedness were selected for non-alcohol-related contexts (mean score for pencil = 1; basket = 1), and the two pictures with the highest ratings were selected for alcohol-related contexts (beer = 4.65; bottles = 4.05). The emotional level was similar among the images with “middle scores”, i.e. not pleasant or unpleasant (pencil = 4.95; basket = 5.05; beer = 5; bottles = 5.25) (Fig. 1).

### Questionnaires

Alcohol craving was assessed before and after the experiment on a visual analogue scale: participants were asked to place a vertical mark through a 100-mm horizontal line on the point they felt best represented their current state of craving. The score was then defined as the distance from the left end of the line to the mark (in mm).

The Alcohol Use Disorder Identification Test (AUDIT) was used to evaluate participants as regard to hazardous drinking, harmful drinking or alcohol dependence [36]. Hazardous drinking, which is of public health significance despite the absence of any current disorder in the individual user, is defined as a level of alcohol consumption likely to result in harm to the user or other individuals [37]. Harmful drinking was defined as alcohol consumption resulting in serious effects on physical, mental and potentially social health [38]. Alcohol dependence is defined as a cluster of behavioral, cognitive and physiological phenomena that may develop after repeated alcohol use and that typically include a strong desire to drink alcohol, difficulties in controlling its use, persistent drinking despite harmful consequences, a higher priority given to drinking than to other activities and obligations, increased alcohol tolerance, and sometimes physical withdrawal [38].

The Urgency Premeditation Perseverance and Sensation seeking impulsive behavior scale (UPPS) [39] is a well-validated and frequently used questionnaire, which describes the difficulty to restrain behavioral reactions in situations that elicit strong emotion (Urgency), the difficulty to anticipate expected situations (lack of Premeditation), the difficulty to sustain prolonged activity (lack of Perseverance), and the tendency to search for new emotionally-arousing situations (Sensation seeking) [40].

Finally, participants were asked to fill out questionnaires assessing psychological measures: the State-Trait Anxiety Inventory (STAI) to assess state and trait anxiety, and the Beck Depression Inventory (BDI) to assess depression [41]–[42]. Indeed, young drinkers with depression and anxiety symptoms have been shown to be at increased risk of alcohol use disorder during young adulthood [43].

### Procedure

After signing the informed consent document and before starting electroencephalogram (EEG) recording, all participants were assessed with the AUDIT, BDI, STAI, UPPS and craving scales. A block of pictures was shown first to familiarize the subjects with the experimental environment and the task ahead. This “practice block” consisted of 12 trials (eight Go and four No-Go) on a black background. Subjects were allowed to rest between blocks. After the task, craving was assessed once again. Finally, each participant was asked to evaluate each picture for alcohol-relatedness, valence and arousal level.

### EEG recording

Electric brain potentials were recorded from 32 electrodes mounted on a Quik-Cap and placed in standard (based on the 10–20 system) and intermediate positions. A common physical linked mastoids reference was used, and the data were later re-referenced to the average value of this common reference [44]. The EEG was amplified by battery-operated amplifiers with a gain of 30,000 and a bandpass of 0.01–100 Hz (Advanced Neuro Technology - ANT Ltd, Enschende, the Netherlands). The ground electrode (AFz) was positioned between Fpz and Fz along the midline, and the impedance of all electrodes was maintained below 10 kΩ. The EEG was recorded continuously at a sampling rate of 1024 Hz with the ANT Eeprobe software.

Approximately 16% of trials were contaminated by eye movements or muscular artifacts, which were manually eliminated offline using the procedure developed by Semlitsch *et al.*, which consists of subtracting an average artifact response for each individual participant based on a percentage of the maximum eye movement potential (generally recorded on Fp1, Fpz and Fp2 prefrontal electrodes) [45]. An ANOVA 3×2, imputing context (A, NA, NC) as within-subject variable, and group (low and high drinkers) as between-subjects variable, showed that the number of rejected trials was similar in each group and condition (context: p = 0.183; group: p = 0.124; group×context: p = 0.808). Epochs were created from −200 to 800 ms after stimulus onset (−200 to 0 considered as the prestimulus baseline). Data were filtered with a 30 Hz low-pass filter.

### Data collection

Three stimulus parameters were recorded: (1) type (Go or No-Go); (2) context (NC or AC or NAC); and (3) response (keypress to Go stimuli or no keypress to No-Go stimuli).

Moreover, for each subject and context, the maximum peak amplitude and latency to peak amplitude of the Go and No-Go N2 and the Go and No-Go P3 components were recorded. The component values were measured with frontocentral electrodes (F3, Fz, F4, FC1, FC2, Cz) about 180–350 ms after stimulus onset for the N2, and 270–590 ms for the P3 [5], [9].

### Statistical analysis

ERP data were analyzed with repeated measures by ANOVA. Simple effects were explored and interaction sources were systematically examined. Students't-tests, ANCOVA, Bonferroni's post-hoc test and Pearson's correlation were used when appropriate. All analyses were done with SPSS 17.02® and the level of significance was set at 0.05.

Omission error rates (i.e., no response in Go trials), commission error rates (i.e., keypress in No-Go trials), and reaction time (RT) to Go stimuli were also analyzed. Omission and commission errors were divided by the number of trials and converted to percentages.

## Results

### Behavioral data

**Error rates:** We investigated participants' performance under three different conditions: neutral (NC) and non-neutral (AC and NAC). To examine whether the AUDIT, UPPS and craving scores had an influence on subjects' performance, an ANCOVA was performed on error rates, imputing trial type (Go and No-Go) and context (NC, AC, NAC) as within-subject variable, and AUDIT, UPPS and craving scores as covariates. As expected, a significant effect of the trial type was observed: error rates were higher for No-Go (commission errors) than Go trials (omission errors) (19% *vs.* 4%, p<0.001). Interestingly, a significant effect of AUDIT (p = 0.006) and craving (p = 0.004) could be observed. Also, there was a significant interaction between trial type and the AUDIT (p = 0.005) but not UPPS score, as well as between trial type and craving (p = 0.003). To clarify these interactions, the participants (n = 35) were divided into two groups, split at the median of AUDIT values: low drinkers (AUDIT≤11; n = 18) and high drinkers (AUDIT>11; n = 17). High drinkers had higher craving scores than low ones, but similar impulsivity scores (Table 1). This way, we could investigate the influence of AUDIT and craving on the Go/No-Go performance. It is important to note that the two groups did not differ in terms of age, gender, anxiety and depression. An ANOVA on error rates was then computed separately for Go and No-Go trials, imputing context as within-subject variable and group (low and high drinkers) as between-subjects variable. Considering Go trials, there was no significant difference between NC, AC and NAC, and no interaction between group and context (p = 0.880). However, considering No-Go trials, there was a trend to interaction between group and context (p = 0.082; ή^2^ = 0.074; Power = 0.492). Indeed, post-hoc tests showed that there was no difference in inhibitory response in NC (18% *vs.* 20%, p = 0.465; Cohen's *d* = −2.61; Effect size *r = −0.79*) and NAC (19% *vs.* 22%, p = 0.417; Cohen's *d* = −3.25; Effect size *r = −0.85*), but a significant difference in AC, as low drinkers made fewer commission errors than high drinkers (15% *vs.* 22%, p = 0.039; Cohen's *d* = −7.59; Effect size *r = −0.96*). The omission and commission error rates are presented in Table 2.

**Table 1:** Characteristics of the study participants*.

	Light drinkers (n = 18)	Heavy drinkers (n = 17)	p-value
Male gender	10 (55.6%)	8 (47.1%)	0.615
Age (years)	21.55 (±2.09)	21.00 (±1.87)	0.415
AUDIT	6.50 (±3.5)	16.90 (±4.8)	<0.001
Number of alcohol doses per week	5.78 (±7.90)	20.92 (±11.25)	<0.001
Hazardous drinking	4.50 (±2.00)	8.88 (±1.57)	<0.001
Alcohol dependence	0.77 (±0.87)	3.70 (±2.41)	<0.001
Harmful drinking	1.27 (±1.31)	4.35 (±2.57)	<0.001
BDI	2.77 (±2.81)	3.41 (±2.37)	0.478
STAI trait	42.72 (±9.76)	46.47 (±8.93)	0.246
STAI state	43.55 (±6.94)	45.64 (±7.11)	0.385
UPPS	98.44 (±9.88)	107.29 (±20.34)	0.119
Craving before experiment	0.41 (±0.78)	2.53 (±2.49)	0.005
Craving after experiment	0.51 (±0.81)	2.42 (±2.37)	0.004

* Mean scores (± standard deviation) of light and heavy drinkers depending on alcohol drinking characteristics.

*BDI: Beck Depression Inventory; STAI: State and Trait Anxiety Inventory; UPPS: Impulsive Behavior Scale; One dose represents 10 g of alcohol.*

**Table 2:** Reaction time to Go stimuli and error rates*.

	Neutral		Alcohol-related		Non-alcohol-related	
	LD^1^	HD^2^	LD^1^	HD^2^	LD^1^	HD^2^
Reaction time (ms)	331 (±27)	314 (±70)	333 (±30)	329 (±51)	328 (±25)	304 (±52)
Omission error (%)	2 (±5)	6 (±6)	3 (±4)	7 (±6)	5 (±5)	10 (±14)
Commission error (%)	18 (±0.6)	20 (±0.9)	15 (±0.7)†	22 (±1.1)†	19 (±0.7)	22 (±1.1)

1 Light Drinkers.

2 Heavy Drinkers.

* Mean reaction time (± standard deviation) to Go stimuli and commission error rates (in %) of light and heavy drinkers in each context (neutral, alcohol-related and non-alcohol-related).

† Statistically significant at p = 0.039.

**Reaction time:** The reaction time in Go trials was analyzed by ANOVA, imputing context as within-subject variable and group (low and high drinkers) as between-subjects variable. The group did not influence RT to Go stimuli, as there was no difference between the two groups (331 ms *vs.* 314 ms, p = 0.354). Moreover, there was no influence of context, as the RT to Go stimuli did not differ between the two groups in NC, AC and NAC (p = 0.537) (Table 2).

Overall, as expected from the literature [31], all participants made more errors in No-Go than Go trials. Moreover, considering No-Go trials, pictorial contexts had an influence on task performance for participants with a high AUDIT score. Finally, the reaction time to Go stimuli was not affected by the context, nor by alcohol consumption.

### ERP data

ERPs have the potential to detect even minor neurocognitive restrictions [46]. To investigate whether alcohol drinking, craving and/or impulsivity affected the potentials elicited by the task, 2×3×6×2 ANCOVA were computed for the N2 and P3 components, imputing trial type (Go/No-Go), context (NC, AC, NAC), electrode (F3, Fz, F4, FC1, FC2 and Cz) and parameters (amplitude, latency) as within-subject variables, while AUDIT, UPPS and craving scores were used as covariates.

**P3 component:** The ANCOVA revealed that AUDIT scores, but not UPPS (p = 0.209) and craving (p = 0.726) scores, tended to reach significance (p = 0.076, ή^2^ = 0.098, Power = 0.428). Moreover, only AUDIT showed a significant interaction with Parameters (p = 0.043). Therefore, to investigate the influence of AUDIT on both P3 parameters, 2×3×6 ANCOVAs were separately performed on P3 latencies and amplitudes.

For P3 latencies, only a main effect of AUDIT was detected (p = 0.05), while the interaction type×context×AUDIT tended to be significant (p = 0.085, ή^2^ = 0.074, Power = 0.493). To study more deeply this trend to interaction, the participants (n = 35) were divided into two groups, split at the median of AUDIT values [low drinkers (AUDIT≤11, n = 18) and high drinkers (AUDIT>11, n = 17)]. Then, ANOVAs 3×6, imputing context and electrodes as within-factors and group (low, high drinkers) as between-subjects variable, were computed for Go and No-Go trials, respectively. While no difference emerged for Go trials (all p>0.100), a main effect of Context (p = 0.006) and an interaction context×AUDIT (p = 0.045) were observed for No-Go trials. Post hoc tests showed that whereas P3 latency was longer for AC than NAC (433 (s.d.: 52) ms *vs.* 407 (s.d.: 34) ms, p = 0.04) in the group of high drinkers, there was no significant difference between the two contexts in the group of light drinkers (416 (s.d.: 19) ms *vs.* 424 (s.d.: 25) ms, p = 0.216). Interestingly, the P3 latency was similar when low and high drinkers were compared for A (416 vs. 433 ms, p = 0.227; Cohen's *d* = −0.43; Effect size *r = −0.21*) and NA (407 vs. 424 ms; p = 0.099; Cohen's *d* = −0.56; Effect size *r = −0.27*).

Regarding P3 amplitudes, no main effect on AUDIT, UPPS and craving was noted (all p>0.300). However, a main effect of Type was observed (p = 0.004), suggesting that P3 amplitudes were higher for No-Go than for Go trials (12.79 (s.d.: 5.38) µV *vs.* 7.96 (s.d.: 2.64) µV; p<0.001). Moreover, there was a significant interaction between type, context and UPPS scores (p = 0.042). To clarify this interaction, the participants (n = 35) were divided into two groups, split at the median of UPPS values [low impulsivity (UPPS≤102, n = 18) and high impulsivity (UPPS>102, n = 17)]. Then, paired t-tests showed that in Low Impulsive participants, No-Go trials generated higher P3 amplitudes than Go trials in each context (A: 14.7 (s.d.: 5.29) µV vs. 8.4 (s.d.: 3.76); p<0.001; NA: 13.6 (s.d.: 6.78) vs.6.6 (s.d.: 4.18); p = 0.003; NC: 12.6 (s.d.: 5.45) vs. 7.8 (s.d.: 3.95); p<0.001). However, only the A context revealed similar results in High impulsive subjects (12.9 (s.d.: 5.89) *vs.* 8.8 (s.d.: 3.43); p = 0.001; while NA: 11.6 (s.d.: 5.85) *vs.* 8.2 (s.d.: 4.65); p = 0.118; NC: 10.9 (s.d.: 6.64) *vs.* 7.8 (s.d.: 5.38); p = 0.062).

**N2 component:** Since some differences had been noted regarding the P3 component, we wondered whether these P3 modulations were not due to prior differences in the N2 component. To test this point, a similar 2×3×6×2 ANCOVA with AUDIT, UPPS and craving scores was performed. However, there was no significant interaction between Parameters and AUDIT (p = 0.179), craving (p = 0.590) or UPPS (p = 0.643) scores, in contrary to what was observed with P3. Therefore, only the higher amplitude for No-Go as compared to Go trials could be replicated with N2 values (−1.044 µV *vs.* 1.717 µV; p<0.001).

**Figure 2:**
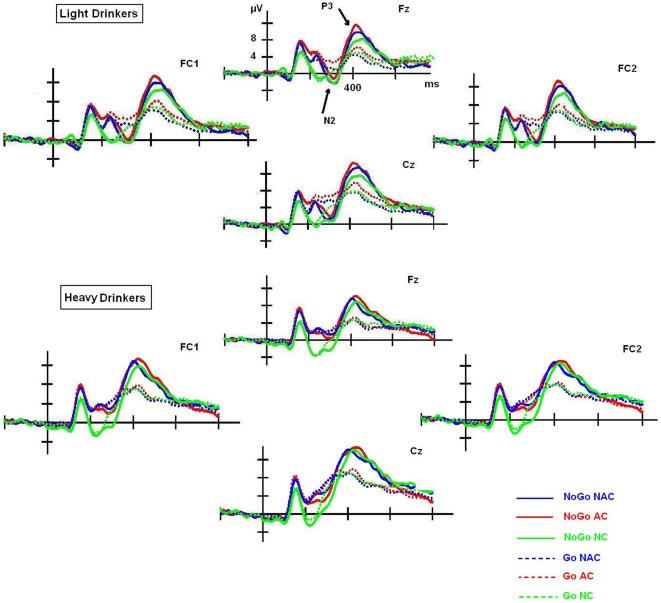
N2 and P3 amplitude. Higher N2 and P3 amplitude in No-Go than Go trials measured on frontocentral electrodes (FC1, Fz, FC2, Cz) in each context (NC, AC and NAC), and in light and heavy social drinkers.

**Figure 3:**
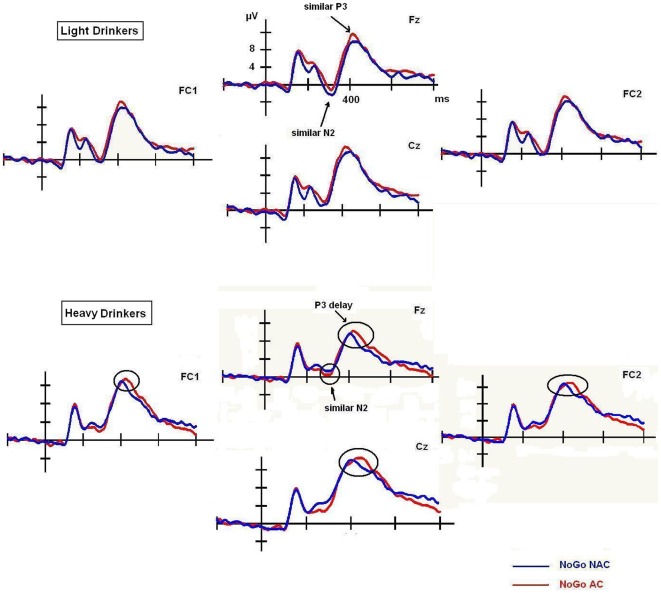
N2 and P3 latency. N2 and P3 recorded for No-Go trials in AC and NAC. No difference was observed with N2. However, heavy drinkers displayed a delayed No-Go P3 in response to AC trials, while light drinkers did not.

Overall, these results showed that N2 and P3 amplitude was associated with inhibitory response. Indeed, the amplitude was larger in No-Go than Go trials in both cases (Fig. 2). Strikingly, the No-Go P3 component, but not the No-Go N2 component, seemed to be modulated by AC only in high drinkers, as this group showed more delayed latency than low drinkers (Fig. 3). Finally, although the AUDIT and craving scores did not affect N2 and P3 amplitudes, highly impulsive participants displayed significant higher P3 amplitudes to No-Go trials than Go trials, but only in AC. However, low impulsive subjects displayed significant higher P3 amplitudes to No-Go trials than Go trials in each context.

### Evaluation of pictures

Table 3 shows the evaluation of each picture by low and high drinkers for alcohol-relatedness, valence and arousal level. Heavy drinkers gave a higher arousal score than light drinkers for alcohol-related pictures (5.32 *vs.* 4.42, p = 0.033; Cohen's *d* = 0.65; Effect size *r = 0.31*), specifically for the picture showing someone drinking a beer (4.16 *vs.* 5.17, p = 0.046; Cohen's *d* = −0.70; Effect size *r = −0.33*). This result is not surprising, as 94% of heavy drinkers reported beer as their preferred and most consumed alcoholic beverage.

**Table 3:** Picture assessment by light and heavy drinkers.

	Light drinkers	Heavy drinkers	p-value
	**Alcohol-relatedness**		
Picture 1- Non-alcohol-related (pen)	1.05 (±0.23)	1.05 (±0.24)	0.968
Picture 2 - Alcohol-related (alcohol bottles)	4.88 (±0.47)	4.88 (±0.33)	0.962
Picture 3 - Alcohol-related (beer)	4.77 (±0.42)	4.58 (±0.61)	0.303
Picture 4 - Non-alcohol-related (basket)	1.05 (±0.23)	1.11 (±0.48)	0.638
	**Valence**		
Picture 1- Non-alcohol-related (pen)	5.50 (±1.09)	5.29 (±0.91)	0.551
Picture 2 - Alcohol-related (alcohol bottles)	4.33 (±1.49)	5.11 (±1.65)	0.152
Picture 3 - Alcohol-related (beer)	5.22 (±1.76)	5.58 (±1.50)	0.513
Picture 4 - Non-alcohol-related (basket)	5.55 (±1.38)	5.23 (±1.48)	0.513
	**Arousal**		
Picture 1- Non-alcohol-related (pen)	4.33 (±1.68)	4.58 (±1.27)	0.616
Picture 2 - Alcohol-related (alcohol bottles)	4.66 (±1.49)	5.47 (±0.94)	0.066
Picture 3 - Alcohol-related (beer)	4.16 (±1.54)	5.17 (±1.33)	0.046
Picture 4 - Non-alcohol-related (basket)	3.55 (±1.75)	3.82 (±1.91)	0.669

Mean (± standard deviation) assessment of each picture by light and heavy drinkers according to alcohol-relatedness (1: non-alcohol-related; 5: alcohol-related), valence (1: negative; 9: positive) and arousal level (1: excited; 9: sleepy).

### Correlations

Pearson correlations were used to test the hypothesis that No-Go scores in AC are associated with the effect of pictures. The results showed that the higher the arousal level of the alcohol-related scene, the higher the number of commission errors in AC (r = 0.402; p = 0.017). However, the arousal generated by non-alcohol-related pictures was not significantly correlated with the rate of errors (pen: r = 0.003; p = 0.988; basket: r = 0.055; p = 0.755). Pearson's correlations were also used to test whether some association between AUDIT, UPPS and craving scores, and commission errors and ERP parameters (N2, P3 latencies and amplitudes) could be detected. Our results showed that alcohol dependence was positively correlated with commission errors in AC (r = 0.33; p = 0.05), when specific items from the AUDIT related to impaired control over drinking (r = 0.404; p = 0.016) were considered. Moreover, a negative correlation with No-Go P3 amplitude in NAC in subjects with high UPPS scores (r = −0.508; p = 0.037), particularly when the Urgency dimension was taken into account (r = −0.635; p = 0.006). Finally, in the group of heavy drinkers, craving was negatively correlated with commission errors in each context (NC: r = −0.594; p = 0.012; A: r = −0.531; p = 0.028; NA: r = −0.579; p = 0.015).

## Discussion

Data from the literature shows that heavy social drinkers (as compared to light drinkers) have a higher alcohol-related cue-reactivity and altered inhibitory processes. These differences are generally reflected by P3 amplitude modulation in No-Go conditions [5], [9], [24]–[26]. In the present study, we showed that heavy drinkers display more commission errors than light drinkers in AC at the behavioral level (15 *vs.* 22%). This observation suggests that when placed in a cue-reactivity context, patterns of alcohol consumption modulate performance in an inhibitory task. Interestingly, Table 2 shows that among heavy drinkers, the rate of commission errors is stable among different contexts (between 20–22%), while light drinkers present less commission errors in AC than in NAC and NC (15% *vs.* 18–19%). This may reflect that, in high drinkers, the alcohol effect affects inhibition performance in all contexts. However, light drinkers are less inclined than heavy drinkers to be cue-reactive in AC, and their general error rate is thus lower. At the neurophysiological level, a delayed No-Go P3 component was found in heavy drinkers as compared to light drinkers, when participants were confronted with a visual Go/No-Go task, performed on an alcohol-related contextual background (as compared to NAC). Although the amplitude was not affected, we associated this No-Go P3 delay with the fact that light drinkers make fewer commission errors than heavy drinkers (in AC compared to NAC). We propose that this latency effect, combined with preserved amplitude, has a double origin. Indeed, some studies have shown that when participants are confronted with dual tasks, the second task induced P3 amplitude decrement *but also longer P3 latency* than the first task, because of the processing priority combined with the limited availability of processing resources [47]. Therefore, if P3 latency reflects stimulus evaluation time, we propose that high drinkers have delayed No-Go P3 in AC (433 ms) as compared to NAC (407 ms). Indeed, their higher reactivity to the alcohol-related background decreased the amount of attentional resources available to perform the task, whereas light drinkers behaved in a similar way in both contexts (416 *vs.* 424 ms). In other words, when confronted to the same task than light drinkers, high drinkers prioritize the processing of the alcohol-related background, and the Go-No-Go task becomes a “secondary” task, leading to specifically longer P3 latencies. But then the question remains as to why there was no decrease in P3 amplitude. A potential explanation for this the absence of amplitude effect may be the sample selection. Indeed, the participants were all university students, and some of them were excessive drinkers (high drinkers). However, recent ERP studies [48]–[49] showed that the influence of alcohol quantity in university students was restricted to a decreased in neuronal processing speed. Several degenerative brain diseases are known to cause abnormalities restricted to the latency of ERP components, such as brain infarction, which slightly delays the P3 latency without affecting its amplitude or scalp distribution [50]. Anatomically, this variability in P3 latency is mainly related to white matter connectivity, also known to be altered by alcohol consumption [51]. The authors suggested that these abnormal delayed latencies could represent a first step before extending to ERP amplitude values (indexing the intensity of information processing; [46]), as in chronic alcoholism [52]. Therefore, further studies should investigate if the latency effect observed in this study could extend to amplitude values when university students (with higher and/or longer alcohol consumption habits) or chronic alcoholic patients are considered.

These results are very important, because they could constitute “a first level of deficit” towards that found in alcohol-dependent patients (generally presenting P3 modulations in terms of both amplitude and latency). Indeed, alcoholics have been shown to preferentially process alcohol-related rather than non-alcohol-related cues, and to present difficulties inhibiting prepotent responses [10], [17], [53]. Moreover, this higher sensitivity/reactivity to alcohol cues and this inhibitory deficit are supposedly key predicting factors (automatic *vs.* controlled) for alcohol relapse following treatment [16], [19]. Therefore, considering that: (1) young drinkers express minimal motivation to remain abstinent [19], (2) automatic processes eliciting heightened attention and triggering motivational orientation can be amplified by drinking alcohol [54], and (3) repeated alcohol exposure interferes with brain maturation and causes lifelong diminished self-controlled regulation, it is clear that social drinking in young drinkers such as university students should be considered a research priority to provide new information for prevention and treatment. In this regard, a delayed No-Go P3 component, observable only on alcohol-related contexts, may be considered in social heavy drinkers as a vulnerability factor in developing alcohol misuse. Therefore, prevention programs aimed at reducing the high arousal of alcohol stimuli and strengthening cognitive control should be developed [5].

Moreover, it is important to note that in our study, several different variables such as age, gender, depression and anxiety were similar between low and high drinkers. Therefore, the correlation found in high drinkers between the arousal generated by alcohol-related pictures, impaired control over drinking and number of commission errors, reinforce the idea that higher alcohol cue-reactivity may lead to lower inhibitory capacity, and thus poorer performance. Interestingly, a negative correlation was also found between craving and commission errors, independently of the background context. This is consistent with the idea that if craving may be associated with poorer inhibitory performance [55], self-reported craving is not a significant predictor of post-drinking behavior, in contrary to cue-elicited brain responses [56]. Indeed, a robust finding in the literature is that heavy drinkers present an increased reactivity to alcohol cues, although there are some individual differences in cue-elicited and self-reported craving measures [57]. In the present study, we showed that craving was higher in high than low drinkers (both before and after the experiment), but that this self-reported craving was associated among heavy drinkers with less commission errors, even if participants were confronted with contexts inducing cue-reactivity. This quite unexpected result should be deeply investigated in further studies. In this regard, personality factors could be involved in the interaction between cue-reactivity and subjective craving, and impulsivity has been proposed as a potential candidate [58]. Here, impulsivity was similar in low and high drinkers. Thus, it did not influence commission errors and the No-Go P3 latency effect found in AC. However, we observed that high impulsive participants displayed higher P3 amplitudes to No-Go trials (as compared to Go ones) only in AC, and independently of AUDIT and craving scores. Moreover, the most impulsive the subjects (particularly regarding the Urgency dimension), the lower the amplitude to No-Go trials was in NAC. This suggested that impulsive personality traits modulated the neural response to alcohol-related and non-alcohol-related cues [34], and that this should be taken into account in all studies investigating cue-reactivity and craving to alcohol.

Finally, we would like to discuss the fact that only P3 was affected in studies investigating cue-reactivity and inhibition in alcohol drinkers [9]–[11]. Indeed, the N2 component, which is thought to reflect conflict monitoring and effortful processing through the activity of the anterior cingulate cortex (ACC), was not affected [6]–[8]. This absence of alcohol effect on N2 may be due to the fact that N2 and error-related negativity (ERN) reflect the involvement of the ACC in conflict monitoring, and that the amplitudes of these two components do not necessarily correlate [59]. For instance, Ridderinkhof et al. found that alcohol consumption led to a substantial reduction of the ERN amplitude, but did not affect the N2 [60]. In other words, dissociations are possible because these two components are sensitive to different aspects of task processing: ERN is related to the ability to produce an error-correcting response, while N2 is related to the processing of irrelevant information, which determines the level of incorrect response activation [59]. Then, the absence of N2 effect in our study could be due to the fact that this component does not tap into the monitoring process itself. Further studies, should investigate whether ERN amplitude and latency could be more affected when participants have to inhibit a response in an alcohol-related context, for example when increasing task difficulty.

Clearly, this study presents some limitations. The main limitation is probably the sample size, which prevented us from detecting highly statistically significant interactions. Moreover, we focused on university students in a specific social context of displaying their alcohol habits. Finally, because of the experimental design (Go/No-Go task), we focused on N2 and P3 components [5], [9], while other ERP studies using different tasks have shown earlier modulations related to alcohol-cue reactivity (e.g., on the visual P1 component) [61]. Therefore, these data should be considered as preliminary, and further studies on larger samples should investigate different ERP components with different tasks, and select drinkers with more heterogeneous patterns of drinking.

Finally, we would like to stress that the methodology of this study was original. Indeed, most studies published separately assessed alcohol-related and non-alcohol-related contexts to investigate whether heavy drinking is associated with higher alcohol cue-reactivity and poorer performance. In this study, we confronted our participants with a Go/No-Go task superimposed on an alcohol-related background, and showed specific impairment in heavy drinkers compared with a neutral or non-alcohol-related context. Therefore, this experimental design implicates more complex behavioral and neural reactions, and may help investigate more precisely the interactions between long-lasting exposure to alcohol-related cues and altered inhibitory capacities [31].

In conclusion, to our knowledge, this is the first study to use a dual Go/No-Go task/contextual pictures methodology with ERPs. This study is preliminary and should be replicated in larger samples of social drinkers. Moreover, further studies should be performed with this methodology to investigate whether a greater inhibitory deficit in in long-term alcohol-related context may be associated with relapse in alcohol-dependent patients. Finally, these experimental conditions may also be applied to other drug-related behaviors, by changing the background pictures.
